# Surveillance and Prediction of Risk Factors for Central Line-Associated Bloodstream Infections in Saudi Arabia

**DOI:** 10.7759/cureus.62699

**Published:** 2024-06-19

**Authors:** Reham Kaki, Abdullatif Zatar, Nuha A Nabalawi

**Affiliations:** 1 Internal Medicine and Infectious Disease and Infection Control, King Abdulaziz University Hospital, Jeddah, SAU; 2 Internal Medicine, King Abdulaziz University Hospital, Jeddah, SAU; 3 Microbiology and Parasitology, King Abdulaziz University Hospital, Jeddah, SAU

**Keywords:** length of hospital stay, infection, central line, clabsi, bloodstream infection

## Abstract

Background: Although central line-associated bloodstream infection (CLABSI) is the most common type of healthcare-associated infection among patients with inserted devices, few studies have comprehensively evaluated the related risk factors.

Objective: This retrospective study analyzed the risk factors, predictors, causative organisms, and impact of CLABSI on clinical outcomes mortality, and length of stay (LOS) in older adults.

Methods: We included 36 patients diagnosed with CLABSI according to the Centers for Disease Control and Prevention criteria at King Abdulaziz University Hospital during 2013-2014 cases and 375 control patients controls. Risk factors were evaluated using a multivariate logistic regression analysis.

Results: Cases and controls did not differ significantly in age or sex distribution. However, cases had a significantly longer LOS than controls 78 vs. 19 days, p < 0.001. One-third of 12/36 CLABSI cases were admitted to the medical intensive care unit (MICU). Most had renal disease, acute coronary syndrome, and used steroids. Additionally, 34 cases (94.4%) and 2 cases (5.6%) presented with primary and secondary infections, respectively, and hypotension was the most prevalent symptom (12/36). The internal jugular vein was the most common insertion site, and the nasogastric tube and mechanical ventilator were the most common insertion devices. Seven cases died, and three deaths were attributed to bloodstream infection (BSI). The most common cause of blood infection was *Staphylococcus epidermidis*, followed by *Klebsiella pneumoniae*.

Conclusions: The present study reveals age, LOS, total parenteral nutrition/partial parenteral nutrition (TPN/PPN), and transplantation as the independent risk factors/predictors of CLABSI.

## Introduction

Healthcare-associated infections, which affect approximately 5-10% of all hospitalized patients, constitute a major safety risk. Approximately 100,000 patients die each year from hospital-acquired infections [1]. Cases of healthcare-associated infections can be classified into four main types: catheter-associated urinary tract infection, central line-associated bloodstream infection (CLABSI), ventilator-associated pneumonia, and surgical site infection [2]. For many patients, treatment and management rely on central line placement, and although these devices enhance vascular access and thus enable the administration of multiple therapies, patients with these devices face an increased risk of infection [3]. Such healthcare-associated bloodstream infections (BSIs) are associated with high rates of mortality and morbidity as well as substantial increases in the duration of hospitalization [4]. Specifically, those who require long-term venous access at baseline face a higher risk of developing a BSI compared to those without a central line. Furthermore, the development of a BSI may lead to substantial increases in healthcare costs and mortality risks [5]. Therefore, CLABSIs must be clearly defined and monitored using standard protocols, as a correct diagnosis could avoid the indiscriminate removal of a central line.

Generally, developing countries have not systematically evaluated the situation of CLABSI, and to our knowledge, no studies have focused exclusively on the impact of BSI on older adults, despite increasing population aging. This is surprising because the incidence of BSI has been shown to increase with age, from a rate of 4.47 per 1,000 patients aged 65-74 years to 18.1 per 1,000 patients aged >85 years [6]. Thus, it is important to better understand the epidemiology and outcomes associated with BSI in older adults given the likelihood of an increasing frequency of BSI among older adults over time as well as increases in regulations and reimbursements associated with BSI. This study aimed to identify the risk factors, predictive factors, causative organisms, and impacts of BSI on clinical outcomes, including mortality and length of stay (LOS), of patients at a tertiary hospital facility in Saudi Arabia.

## Materials and methods

Patient selection and data collection

This retrospective study was conducted at King Abdulaziz University Hospital (KAUH) in Jeddah, Saudi Arabia, which has a capacity of 1000 beds. The study included patients diagnosed with CLABSI across all departments of KAUH from January 1, 2013, to December 31, 2014. During this period, 36 patients who developed bacteremia due to central line infections were selected as cases. The inclusion criteria specified patients who had a central line inserted at KAUH and retained it for more than 48 hours post-admission. Only patients with positive blood cultures indicating central line infection were included. Exclusion criteria encompassed cases with positive blood cultures stemming from other sources, such as skin and soft tissue infections, intra-abdominal infections, or urinary tract infections.

Definition of central line infection

Catheter-Related Bloodstream Infection (CRBSI) As per the Infectious Diseases Society of America (IDSA)

CRBSI is diagnosed through one of the following criteria: the same pathogen isolated from a quantitative blood culture drawn through the central line and from a peripheral vein with a single bacterial colony count at least threefold higher in the central line sample compared to the peripheral sample; the same organism recovered from percutaneous blood culture and a quantitative (>15 colony-forming units) culture of the catheter tip; or a shorter time to positive culture (>2 hours earlier) in the central line sample than the peripheral sample (differential time to positivity (DTP)) [7].

CLABSI As per the Centers for Disease Control and Prevention (CDC)

CLABSI is identified through the recovery of a pathogen from a blood culture (a single blood culture for organisms not typically present on the skin and two or more blood cultures for organisms commonly present on the skin) in a patient with a central line at the time of infection or within 48 hours before infection onset. The infection must not be related to any other infection the patient may have and should not have been present or incubating at the time of hospital admission [8].

Patients with a central line who did not develop a BSI served as controls (n = 375). Data extracted from patient records included demographics, comorbidities, renal disease, transplant status, malignancy, ischemic heart disease, site of line insertion, type of device used, mechanical ventilation, presence of other devices (such as nasogastric tube, gastrostomy tube, total parenteral nutrition (TPN)), length of hospital stay, intensive care unit (ICU) admissions, steroid use, microbiology results, and presence of neutropenia (absolute neutrophil count < 500 cells/mm³).

Ethics statement

The authors confirm adherence to the journal's ethical policies as stated in the author's guidelines. Approval was obtained from the Unit of Biomedical Ethics, Research Ethics Committee at King Abdulaziz University.

Microbiological identification

Microbial species were identified using the VITEK MS system (bioMérieux, Marcy-l’Étoile, France) with VITEK MS v4.0 software. Antimicrobial susceptibility testing (AFST) was performed according to the Clinical and Laboratory Standards Institute microdilution method using the Sensititre YeastOne panel (Thermo Scientific, Waltham, United States). Minimum inhibitory concentration (MIC) values were interpreted following the tentative breakpoints proposed by the US Centers for Disease Control and Prevention (CDC, 2022).

Statistical analysis

Data analysis was conducted using IBM SPSS Statistics for Windows, Version 22 (Released 2013; IBM Corp., Armonk, New York, United States). Categorical variables were compared using the chi-squared or Fisher’s exact test and presented as numbers and frequencies (%). Continuous variables were compared using Student’s t-test and presented as means ± standard deviations. For non-normal distributions, the Wilcoxon rank-sum test was used, and data were presented as medians and ranges. Univariate analyses identified factors associated with CLABSI development, followed by logistic regression to determine independent risk factors and predictors of CLABSI, including odds ratios (ORs) and 95% confidence intervals (CIs). All tests were two-tailed, and a p-value < 0.05 was considered statistically significant.

## Results

Table 1 presents data from the 36 patients with CLABSI and 375 controls included in this study. The cases and controls did not differ significantly in terms of age (58 (3-90.11) vs. 54 (1-95) years, p = 0.43). However, the LOS was significantly longer among cases, compared to controls (78 (15-284) vs. 19 (1-250) days, p < 0.0001).

**Table 1:** Demographic and clinical characteristics of cases and controls Age and LOS are presented as median (range). All other variables are presented as numbers (%). The chi-squared test (or Fisher’s exact test if frequency < 5) was used to evaluate differences in frequencies between cases and controls. P-values < 0.05 were considered significant. LOS: length of stay; IJV: internal jugular vein; SCV: subclavian vein; PICC: peripherally inserted central venous catheter; NGT: nasogastric tube; TPN/PPN: total parenteral nutrition/peripheral parenteral nutrition; GI: gastrointestinal; NA: not applicable. ^*^Missing data: site of insertion (2 missing values from cases, frequencies were calculated out of 34 cases) and arterial type (7 missing values).

Variables	Case n = 36	Control n = 375	p
Age	58 (3, 90)	54 (1, 95)	0.43
Sex			
Male	17	189	0.73
Female	19	186	
LOS	78 (15, 284)	19 (1, 250)	<0.001
Site of insertion*			
IJV	20 (58.82)	277 (73.87)	0.06
Femoral	7 (20.59)	69 (18.40)	0.88
SCV	4 (11.76)	29 (7.73)	0.34
PICC	2 (5.88)	0 (0)	NA
Tunneled hemodialysis catheter	1 (2.94)	0 (0)	NA
Type of inserted device			
^*^Arterial	7 (24.14)	0 (0)	NA
^*^Hickman dialysis line	5 (16.67)	69 (18.40)	0.81
Mechanical ventilator	27 (75.00)	72 (19.20)	<0.001
NGT	28 (77.78)	363 (96.80)	<0.001
TPN/PPN	5 (13.89)	17 (4.53)	0.02
Renal disease	20 (55.56)	166 (42.27)	0.19
Morbidities			
Transplant	1 (2.78)	0 (0)	NA
Liver DS	1 (2.78)	37 (9.87)	0.23
Malignancy	3 (8.33)	58 (15.47)	0.33
Acute coronary syndrome	16 (44.44)	111 (29.60)	0.07
Neutropenia (<500)	0 (0)	36 (9.60)	NA
Weight (<5 kg)	1 (2.78)	26 (6.93)	0.49
Gastrostomy	0 (0)	1 (0.27)	NA
Steroid use	12 (33.33)	128 (34.13)	0.93

Of the 36 patients with CLABSI, 17 (47.2%) and 19 (52.8%) were male and female, respectively, but this difference was not statistically significant (p = 0.73). Patients with CLABSI were most frequently admitted to the medical intensive care unit (MICU; 12/36, 33.33%), followed by the pediatric ICU (6/36, 16.67%), surgical ICU (5/36, 13.89%), operating room (4/36, 11.11%), FMW 4/36 (11.11%), neonatal ICU (2/36, 5.56%), catheterization laboratory (2/36, 5.56%) and MSW (1/36, 2.78%) (Figure 1). The internal jugular vein (IJV) was the most common site of insertion (20/34, 58.82%), and a nasogastric tube (NGT; 28/36, 77.78%) and mechanical ventilator (27/36, 75.00%) were the most common insertion devices. Patients with CLABSI were significantly more likely to have used a mechanical ventilator (p < 0.0001) and total/partial parenteral nutrition (TPN/PPN) (p = 0.02) but significantly less likely to have used an NGT, compared to the controls (p < 0.0001). Among patients with CLABSI, renal disease and acute coronary syndrome were the most common morbidities, and a majority of patients used steroid drugs (Table 1). Thirty-four (94.4%) and two cases (5.6%) involved first and second infections, respectively. About 31, 6, and 20 cases had an ICU or other central line, permanent central line, temporary central line, and 1 case had a non-umbilical central line. The most prevalent sign and symptom was hypotension (12/36), followed by hypothermia (2/36) and bradycardia (1/36). Furthermore, seven patients with CLABSI died, and BSI was identified as causative in three of the seven deaths.

Table 2 presents the results of the analysis of potentially causative infectious pathogens. Notably, the most common cause of blood infection was coagulase-negative Staphylococci (CONS; 5/36, 13.9%) followed by *Klebsiella pneumoniae* (4/36, 11.1%). Among the patients infected with CONS, two were admitted to the medical ICU, two were admitted to the pediatric ICU, and one was admitted to the surgical ICU.

**Table 2:** Pathogens identified in cases of central line-associated bloodstream infection (CLABSI) and departments where the infections were acquired CONS: coagulase-negative Staphylococci; ESBL: extended-spectrum beta-lactamases; MRSA: methicillin-resistant *Staphylococcus aureus*; MDR ABB: multidrug-resistant *Acinetobacter baumannii*; VRE: vancomycin-resistant Enterococcus; *E. coli*: *Escherichia coli*; NICU: neonatal intensive care unit; MICU: medical ICU; PICU: pediatric ICU; OR: operating room; SICU: surgical ICU; CATH LAB: catheterization laboratory; MSW: male surgical ward; FMW: female medical ward

Organism	n	%	Location
Klebsiella pneumonia	4	11.1	NICU, PICU, CATH LAB, OR
*Candida albicans* or *Enterococcus faecium*	1	2.8	MICU
Staphylococcus epidermidis	5	13.9	MICU, PICU, SICU
Stenotrophomonas	2	5.6	MICU, OR
ESBL *Klebsiella pneumonia*	3	8.3	MICU, MSW, FMW
*Pseudomonas*/*Acinetobacter*	1	2.8	MICU
Providencia	1	2.8	OR
ESBL *Escherichia coli*	2	5.6	MICU, SICU
MRSA	2	5.6	OR, PICU
Enterococcus faecalis	2	5.6	MICU
MDR ABB	1	2.8	SICU
*Candida krusei*, *Candida albicans*, *Enterococcus faecium*	1	2.8	MICU
*Stenotrophomonas *& VRE	1	2.8	PICU
Candida tropicalis	2	5.6	MICU, SICU
Serratia marcescens	1	2.8	CATH LAB
Candida glabrata	1	2.8	SICU
Stenotrophomonas maltophilia	1	2.8	FMW
*Enterobacter*/*Klebsiella*	1	2.8	FMW
Pseudomonas	3	8.3	MICU, FMW
Acinetobacter baumanii	1	2.8	PICU

Finally, the results of a multivariate logistic regression analysis are presented in Table 3. Here, age (OR: 9.23, 95% CI: 4.17-28.8, p = 0.00), LOS (OR: 34.10, 95% CI: 4.84-34.96, p = 0.00), TPN/PPN (OR: 5.65, 95% CI: 1.05-12.31, p = 0.02, and transplantation (OR: 10.41, 95% CI: 2.04-11.41, p = 0.00) were identified as the independent risk factors or predictors of CLABSI.

**Table 3:** Logistic regression analysis to identify risk factors and predictors of central line-associated bloodstream infection (CLABSI) CI: confidence interval; TPN/PPN: total parenteral nutrition/peripheral parenteral nutrition; GI-DS: gastrointestinal-dysfunction score; LOS: length of stay

Variable	Odds ratio (95% CI)	Significance (p-value)
Sex	0.12	0.73
Age	9.23 (4.17-28.8)	<0.001
LOS	34.10 (4.84-34.96)	<0.001
Hickman dialysis line	0.10	0.76
TPN/PPN	5.65 (1.05-12.31)	0.02
Renal disease	1.65	0.20
Transplant	10.41 (2.04-11.41)	<0.001
Liver disease	1.68	0.20
Malignancy	1.34	0.25
Acute coronary syndrome	3.21 (1.72-8.86)	0.07
Neutropenia < 500	3.57	0.06
Weight < 5 kg	0.93	0.33
GI-DS	0.73	0.39
Steroid use	0.02	0.97

## Discussion

CLABSIs can be acquired at various sites of central line insertion, such as veins in the neck, chest, and groin, and in various contexts, including the provision of medications or fluids or the collection of blood for clinical investigations [9]. Considerable research on the design and evaluation of interventions intended to reduce the risk of CLABSI has yielded progress, as indicated by a 58% decrease in ICU CLABSI rates from 2001 to 2008 in the most recent National Healthcare Safety Network (NHSN) report [10]. Despite these recent successes in the reduction of CLABSI rates in the ICUs of facilities affiliated with the NHSN, significant efforts are needed further to reduce the impacts of these infections on healthcare systems.

Almuneef et al. (2006) previously identified *K. pneumoniae*, CONS, and *Pseudomonas aeruginosa* as the organisms most commonly isolated from patients with catheter-related BSIs [11]. By contrast, we found that CONS was the most frequent causative organism, followed by *K. pneumoniae*. Both types of infection were largely acquired via device insertion into the IJV, suggesting that this site is a major infection route. By contrast, Noaman et al. more recently reported that patients admitted with the co-morbidity of acute renal failure were significantly more likely to develop CLABSI via femoral line access [12-13] reported the duration of ICU central access, central venous catheter placement in the ICU, non-operative cardiovascular disease, presence of a gastrostomy tube, receipt of parenteral nutrition, and receipt of blood transfusion as independent predictors of CLABSI. The study by Timsit et al. found that the use of parenteral nutrition and the presence of gastrostomy tubes significantly increased the risk of CLABSI in critically ill adults. Additionally, chlorhexidine-impregnated sponges and less frequent dressing changes effectively reduced the incidence of CLABSI​ [14]. The study by Hanna et al. identified that blood transfusions and the use of TPN were significant risk factors for CLABSI in critically ill patients. Additionally, the use of antibiotic-impregnated catheters significantly reduced the incidence of nosocomial and multidrug-resistant bacteremias [15]. By contrast, the present study identified age, LOS, TPN/PPN, and transplantation as independent risk factors/predictors for CLABSI.

The study findings indicate that more rapid and accurate methods for diagnosing BSI and CLABSI would improve the timeliness of appropriate clinical patient management, including catheter removal and effective antimicrobial therapy implementation. Optimizing the care of patients with regard to BSI prevention and management presents a major challenge to healthcare providers and administrators. However, this scenario also provides a great opportunity to improve patient healthcare. Ideally, the incidence of CLABSI can be best minimized by maintaining the highest levels of sterile and hygienic conditions in hospitals and removing central lines as soon as they are no longer required. Clinicians and health care providers must adhere strictly to regulations during line insertion to ensure sterile conditions and prevent CLABSI. In addition to appropriate insertion, medical practitioners must ensure stringent sterile conditions when checking the line or changing the dressing. Patients who develop a CLABSI will be febrile and may develop redness and soreness around the central line insertion site. In such cases, medical practitioners and other healthcare providers must examine the patient to determine the presence of contamination [9].

## Conclusions

This study underscores the critical need to address risk factors associated with CLABSI, particularly in older adults within tertiary care facilities. Identifying age, LOS, TPN/PPN, and transplantation as independent predictors highlights the necessity for targeted prevention strategies, especially as the aging population is likely to increase the burden of CLABSI, impacting healthcare costs and clinical outcomes negatively. Enhancing surveillance systems, adopting rigorous infection control practices, and implementing best practices in line management are essential. The role of hospital policymakers and healthcare providers in fostering a safety culture that prioritizes infection prevention is pivotal. By integrating advanced diagnostic tools and focusing on timely and accurate detection, optimizing treatment protocols, and improving patient management, healthcare facilities can significantly reduce the incidence of CLABSI. Continuous education and training of healthcare personnel on catheter insertion and maintenance are crucial. The study advocates for future research focusing on longitudinal tracking to understand long-term outcomes and the effectiveness of various prevention protocols across different hospital settings, with an emphasis on the impact of new technologies and practices in line care and infection monitoring.

The study presents a comprehensive analysis of risk factors, predictors, causative organisms, and the impact of CLABSI on clinical outcomes in older adults, addressing a significant literature gap and providing insights into a particularly vulnerable group. Utilizing robust statistical methods, such as multivariate logistic regression, and adhering to stringent CDC criteria for diagnosing CLABSI, adds to the reliability and validity of the findings. However, the retrospective nature of the study may limit data accuracy and completeness, and being conducted in a single tertiary care hospital in Saudi Arabia may restrict the generalizability of the findings. A larger, multi-center study would be beneficial to validate these results. The study also acknowledges potential biases, such as selection and information biases, which could impact results. Notably, the prominence of *S. epidermidis* as a primary causative organism underlines the need for ongoing surveillance and tailored infection control measures.
